# The complete chloroplast genome sequence of *Vitis berlandieri*

**DOI:** 10.1080/23802359.2020.1797566

**Published:** 2020-07-29

**Authors:** Jiang Xiang, Jianhui Cheng, Lingzhu Wei, Pengfei Cui, Mingshan Li, Jiang Wu

**Affiliations:** Institute of Horticulture, Zhejiang Academy of Agricultural Sciences, Hangzhou, China

**Keywords:** *Vitis berlandieri*, chloroplast genome, rootstock, phylogenetic analysis

## Abstract

*Vitis berlandieri*, a species of grape native to the southern North America, is known for good tolerance against soils with a high content of lime and was almost used for rootstock breeding. Here, we report the complete chloroplast genome of *V*. *berlandieri.* The chloroplast genome was 161,028 bp in length, harboring a large single-copy region (89,228 bp) and a small single-copy region (19,028 bp) separated by two inverted repeat regions. A total of 130 unique genes were identified from this genome, including 85 protein-coding genes (PCGs), 37 tRNA genes, and 8 rRNA genes. Chloroplast phylogenetic analysis revealed that *V*. *berlandieri* is closely related to *V. cordifolia*.

*Vitis berlandieri* was usually used as a breeding partner in rootstock to improve the adaptation of the rootstock to lime soils (Schmid et al. 2009). Many rootstocks currently used in production, such as ‘1103P’ and ‘Richter 110’, were obtained from the cross between *V. berlandieri* and other varieties of rootstocks (Mazzitelli and Schubert 1990; Thomas et al. 2008). However, the phylogenetic relationship between *V. berlandieri* and other *Vitis* species are still uncertain. Thus, we sequenced the complete chloroplast genome of *V. berlandieri* to elucidate its phylogenetic position in the family.

The fresh leaves of *V. berlandieri* were collected from Research Vineyard for Grape Germplasm and Breeding in Zhejiang Academy of Agricultural Science, Xucun Town, Haining City, Zhejiang Province, China (120°24′15″E; 30°26′15″N). Total genome DNA was extracted with the slightly modified CTAB method (Qu et al. 1996). The voucher specimens of *V. berlandieri* were deposited in the Herbarium of the Institute of Horticulture, Zhejiang Academy of Agricultural Sciences (Accession number: ZAAS-XJ-20-06). DNA library was sequenced, and 150 pb paired-end reads were generated on an Illumina NovaSeq 6000 platform (Illumina Inc., San Diego, CA, USA). The 11.9 Gb sequence clean reads were assembled using NOVOPlasty v3.8.3 (Nicolas et al. 2016) with the reference chloroplast genome of *V. vinifera* (GenBank: DQ424856). The chloroplast genome annotation and prediction were conducted using BLAST 2.6.0+ and GeSeq package, respectively (Michael et al. 2017).

The complete chloroplast genome of *V. berlandieri* (GenBank: MT604110) is 161,028 bp in length, and has a typical quadripartite construction, which contains two inverted repeat regions (IRA and IRB) of 52,772 that is insulated by a large single-copy (LSC; 89,228 bp) and a small single-copy region (SSC; 19,028 bp). The total GC content of complete chloroplast genome, LSC, SSC, IR region is 35.32%, 42.95%, 31.64%, respectively. The genome contained a total of 130 unique genes, including 85 protein-coding genes (PCGs), 37 tRNA genes, and 8 rRNA genes. Most of these genes are single copy genes. However, 6 PCGs, 7 tRNAs, and 4 rRNAs have two copies, and only one tRNA (trnM-CAU) has four copies.

To confirm the phylogenetic position of *V. berlandieri* within Vitaceae, the complete chloroplast genomes of 22 other grapevine species were downloaded from the NCBI GenBank database. The sequences were aligned using MAFFT v7 (Katoh et al. 2017), and then the maximum likelihood tree (Figure 1) was constructed by using MEGA X (Kumar et al. 2018). Phylogenetic analysis showed that *V. berlandieri* is closely related to *V. cordifolia*. The published *V. berlandieri* chloroplast genome provides useful information for phylogenetic and evolutionary studies in Vitaceae.

**Figure 1.**
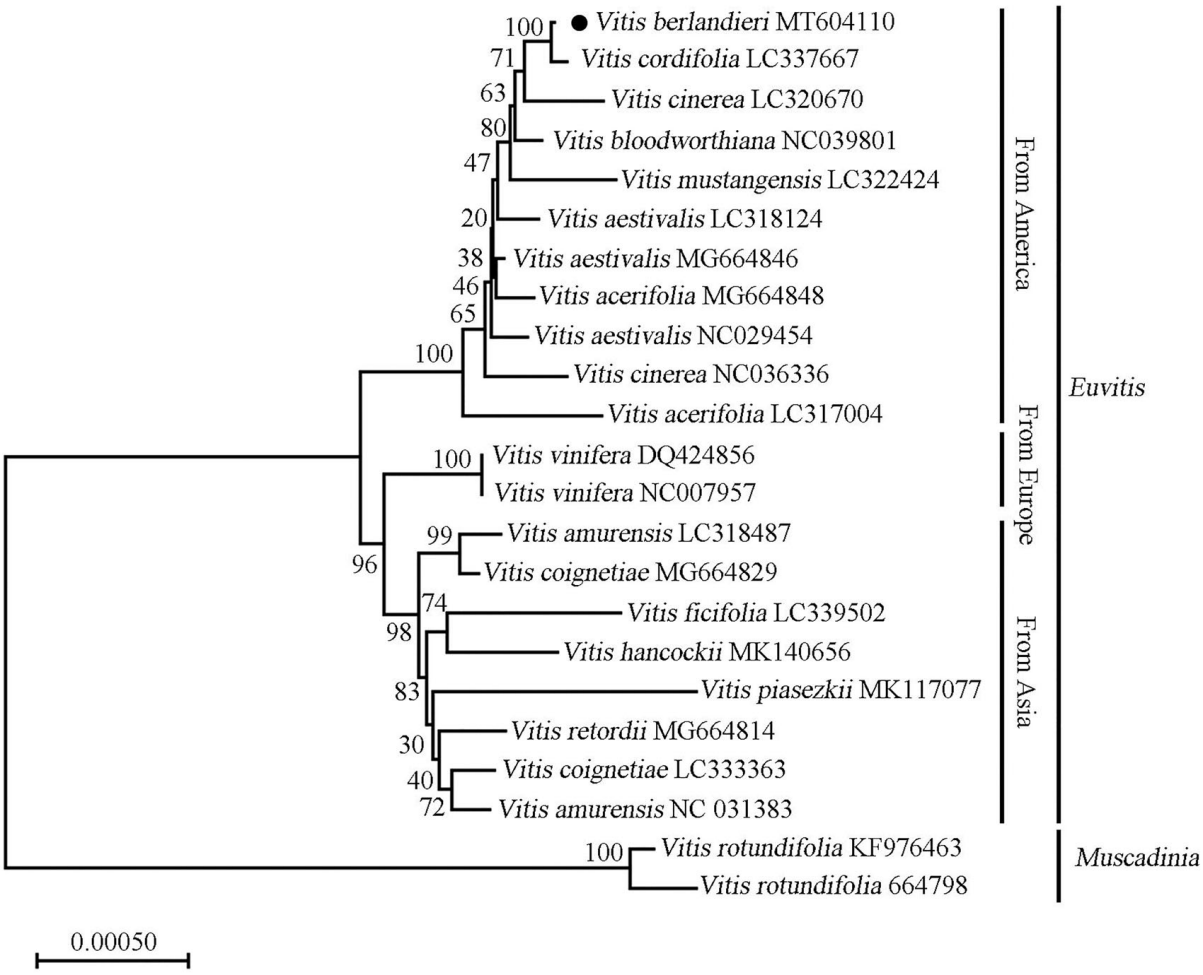
The phylogenetic relationship of 23 species within the *Vitis* species based on neighbour-joining analysis of chloroplast genomes. The bootstrap values were based on 1000 replicates and were shown next to the nodes.

## Data Availability

The data that support the findings of this study are openly available in Genbank at https://www.ncbi.nlm.nih.gov/genbank/, reference number [MT604110], or available from the corresponding author.

## References

[CIT0001] Katoh K, Rozewicki J, Yamada KD. 2017. MAFFT online service: multiple sequence alignment, interactive sequence choice and visualization. Brief Bioinform. 4:1–7. 10.1093/bib/bbx108 PMC6781576 28968734

[CIT0002] Kumar S, Stecher G, Li M, Knyaz C, Tamura K. 2018. MEGA X: molecular evolutionary genetics analysis across computing platforms. Mol Biol Evol. 35(6):1547–1549. 29722887 10.1093/molbev/msy096 PMC5967553

[CIT0003] Mazzitelli M, Schubert A. 1990. Effect of several VAM endophythes and artificial substrates on in vitro propagated *Vitis berlandieri* x *rupestris* “1103 P”. Agric Ecosyst Environ. 29(1–4):289–293.

[CIT0004] Michael T, Pascal L, Tommaso P, Pellizzer T, Ulbricht-Jones ES, Fischer A, Bock R, Greiner S. 2017. GeSeq – versatile and accurate annotation of organelle genomes. Nucleic Acids Res. 45(W1):W6–W11. 28486635 10.1093/nar/gkx391 PMC5570176

[CIT0005] Nicolas D, Patrick M, Guillaume S. 2016. NOVOPlasty: de novo assembly of organelle genomes from whole genome data. Nuclc Acids Res. 45(4):e18. 10.1093/nar/gkw955 PMC5389512 28204566

[CIT0006] Qu X, Lu J, Lamikanra O. 1996. Genetic diversity in muscadine and American bunch grapes based on randomly amplified polymorphic DNA (RAPD) analysis. JASHS. 121(6):1020–1023.

[CIT0007] Schmid J, Manty F, Cousins P. 2008. Collecting *Vitis berlandieri* from native habitat sites. Proc. 2^nd^ Annual National Viticulture Research Conference, July 9–11, 2008. Davis, USA, 73–74..

[CIT0008] Thomas G, Klaus E, Ralph S, Christopher N. 2008. Production and rooting behaviour of *rol*B-transgenic plants of grape rootstock ‘Richter 110’ (*Vitis berlandieri* × *V. rupestris*). Plant Cell Tissue Organ Culture. 94(3):269–280.

